# Optimized Doping of Diffusion Blocking Layers and Their Impact on the Performance of Perovskite Photovoltaics

**DOI:** 10.1021/acsaelm.3c00900

**Published:** 2023-10-12

**Authors:** Fedros Galatopoulos, Sapir Bitton, Maria Tziampou, Nir Tessler, Stelios A. Choulis

**Affiliations:** † Molecular Electronics and Photonics Research Unit, Department of Mechanical Engineering and Materials Science and Engineering, Cyprus University of Technology, Limassol 3603, Cyprus; ‡ Sara and Moshe Zisapel Nano-Electronic Center, Department of Electrical Engineering, Technion-Israel, Institute of Technology, Haifa 32000, Israel

**Keywords:** perovskite solar cells, diffusion mechanisms, fullerenes, doping, conductivity, thermal stability, lifetime, electron-transporting layers

## Abstract

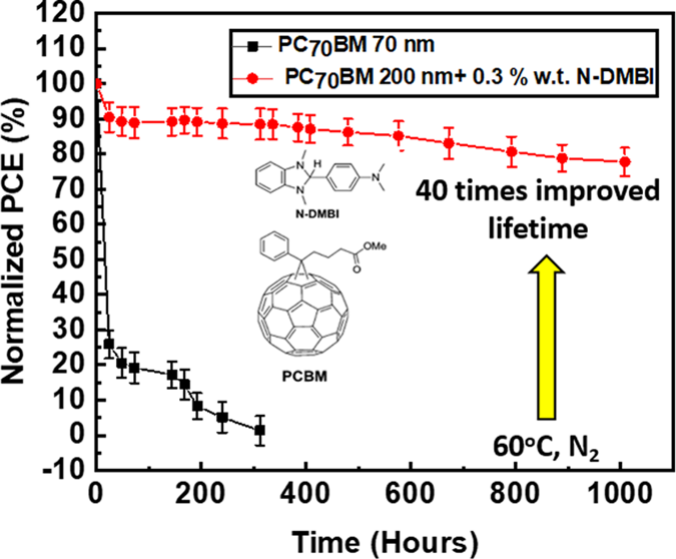

The roll-to-roll printing production process for hybrid organic–inorganic perovskite solar cells (PSCs) demands thick and high-performance solution-based diffusion blocking layers. Inverted (p-i-n) PSCs usually incorporate solution-processed PC_70_BM as the electron-transporting layer (ETL), which offers good electron charge extraction and passivation of the perovskite active layer grain boundaries. Thick fullerene diffusion blocking layers could benefit the long-term lifetime performance of inverted PSCs. However, the low conductivity of PC_70_BM significantly limits the thickness of the PC_70_BM buffer layer for optimized PSC performance. In this work, we show that by applying just enough *N*-DMBI doping principle, we can maintain the power conversion efficiency (PCE) of inverted PSCs with a thick (200 nm) PC_70_BM diffusion blocking layer. To better understand the origin of an optimal doping level, we combined the experimental results with simulations adapted to the PSCs reported here. Importantly, just enough 0.3% wt *N*-DMBI-doped 200 nm PC_70_BM diffusion blocking layer-based inverted PCSs retain a high thermal stability at 60 °C of up to 1000 h without sacrificing their PCE photovoltaic parameters.

## 1 Introduction

Over the past decade, research interest toward developing low-cost photovoltaic (PV) technologies has emerged. Solution-processed hybrid organic–inorganic perovskite solar cells (PSCs) have exhibited a substantial increase in their PCE from 3.8 to 25.7% certified PCE.^1,2^ PSCs offer several advantages and attractive features, such as low exciton binding energy,^3,4^ long carrier diffusion length,^5^ high absorption coefficient,^6^ tunable band gap,^7,8^ and high carrier mobility.^9,10^

Although PSCs showed considerable advancement toward PCE, their long-term stability under high humidity, heat, and illumination conditions of such devices is still a major challenge that needs to be overcome.^11^ The intrinsic stability, under humidity conditions, of the active layer is particularly poor due to the hygroscopic nature of perovskite cations, such as methylammonium (MA^+^), which results in the decomposition of the perovskite crystal under moisture ingress.^12−15^ Decomposition of the perovskite crystal has also been reported under exposure to atmospheric oxygen and ultraviolet illumination, which results in the formation of superoxide ions (O_2_^–^) and the deprotonation of MA^+^ cations. Replacement of the organic cation by inorganic cations such as Cs was adopted as a strategy to improve the stability; however, fully inorganic PSCs are still lacking compared to their mixed halide counterparts in terms of their capability for large-solution-processed PSCs. Furthermore, other drawbacks of the fully inorganic PSC approach are their relatively large band gap and the lower absorption coefficient compared to mixed halide PSCs.^16^ Furthermore, the organic cations incorporated in mixed halide PSCs help in the mitigation of nonradiative recombination as previously reported, providing at this stage at least higher PCE values.^17^ High thermal conditions can affect the stability of PSCs as well, especially when combined with ambient environmental conditions. Heat can promote the diffusion of mobile anions such as I^–^ toward the electrodes of the device as well as promote the diffusion of metal atoms toward the active layer itself.^18−20^ Several approaches were reported to mitigate these effects, such as the incorporation of metal oxides in the electrodes of the device, such as NiO*_X_*^21^ and γ-Fe_2_O_3_,^22^ using a thin Cr layer to isolate the electrodes from the active layer^23^ and utilization of amino acids either as cross-linkers^24^ or for surface treatment and modification of the metal oxide under-layer electrodes.^25,26^ Apart from improved stability, up-scalability of PSCs is also an important factor to consider in order to push PSCs toward commercialization. Even though the aforementioned approaches have successfully improved the stability of PSCs, their incorporation into large-area PSC processing is not straightforward. Solution-processed fullerene buffer layers are an essential component of inverted perovskite PV device architecture, which can be easily upscaled for large-area processing. In our previous work, we showed a simple yet effective method to improve the thermal stability of PSCs utilizing thick fullerene diffusion blocking layers. However, we have also shown that by increasing the thickness of the fullerene layers, there is a dramatic drop of the FF and PCE due to the limited conductivity of the [6,6]-phenyl-butyric acid methyl ester (PCBM) even if the stability is improved.^18^ Our previous experimental results on proposing thick fullerene diffusion blocking layers for long-lived PSCs also verified with the findings of Bitton et al^27^ In their work, they have successfully simulated ionic diffusion in PSCs and have also shown that by increasing the thickness of PCBM from 50 to 200 nm, not only the ionic diffusion is prolonged but also the ionic accumulation at the contacts is significantly reduced, indicating that it will take at least 10 times longer to start observing some degradation by incorporating a 200 nm fullerene-based diffusion blocking layer within the inverted PSC device structure.^27^

Large-scale ETL fabrication techniques, such as Dr. Blade and roll–roll, are often utilized for the fabrication of large-scale PV devices.^28^ Therefore, utilizing thick ETL layers that are compatible with such techniques is desirable for the increased scalability of PSCs. Currently, this is problematic when using PC_70_BM as the ETL for PSCs due to the limited conductivity of the material. One of the most well-known methods to improve the conductivity of PC_70_BM is via doping using n-type dopants, such as 4-(2, 3-dihydro-1, 3-dimethyl-1*H*-benzimidazol-2-yl)-*N* and *N*-dimethylbenzenamine (*N*-DMBI). This doping mechanism follows the general consensus that the dopant functions as a hydride atom donor to the host material (PC_70_BM), followed by an electron transfer step between the molecules of the host material,^29^ due to the natural affinity of PC_70_BM to undergo hydrogenation reactions with various hydrogen atom donors.^30^*N*-DMBI was reported to increase the conductivity of PCBM by 4–6 orders of magnitude (from ∼10^–9^ S/cm to 5 × 10^–3^ S/cm).^31−34^ It has also been shown that PCBM exhibits a decrease of the Fermi level (*E*_F_) from 5.04 to 4.78 eV toward the lowest unoccupied molecular orbital (LUMO), which further highlights the n-type doping of the material.^34^ Therefore, n-type doping of PCBM with *N*-DMBI has been shown to be an effective and beneficial way to increase the conductivity and efficiency of devices, which has been demonstrated in various applications, such as organic photodiodes (OPDs)^31^ and PSCs.^33,34^ It is important to note that doping should usually follow the “just enough” concept, meaning that it should not exceed its optimal value. It has been suggested by Bitton et.al that when charge collection (CL) layers in PSCs are doped excessively, the device performance may degrade.^35^ Here, we revisit that statement and show the negative effect of excess doping on the PSC lifetime. We use the same simulation as in ref (35) to shed light on the mechanism behind such optimal doping.

One of the biggest tasks in the design of high-performance inverted PCSs is the development of high-performance solution-processed diffusion blocking layers. In our previous work, we have shown that thick fullerene buffer layers improve the thermal stability of PSCs; however, the increase in fullerene buffet layer thickness was detrimental to the FF and PCE.^13^ In this work, we have incorporated *N*-DMBI doping in conjunction with a thick (200 nm) PC_70_BM diffusion blocking layer to achieve thermally stable PSCs without sacrificing their photovoltaic (PV) parameters and PCE. In particular, using an optimum doping of 0.3% wt, we managed to improve the *J*_SC_ from 15.2 to 19 mA/cm^2^, FF from 52.6 to 70.3%, and PCE from 7.84 to 13.1% when comparing thick (200 nm) undoped and doped PC_70_BM-based devices while still retaining good thermal stability with *T*_80_= 1000 h (time in which the PCE reached 80% of its initial value) under accelerated heat conditions (60 °C, N_2_ atmosphere). The improvement of *J*_SC_ and FF is directly correlated to the increase in conductivity of PC_70_BM, reduction of series resistance (*R*_S_) from 13.1 to 1.5 Ω, and increase of shunt resistance (*R*_SH_) from 41.7 to 50.2 kΩ. The optimum doping density is correlated to the iodide deficiency in the perovskite layer through the presented simulations.

## 2 Results and Discussion

### 2.1 Solar Cell Device Structure and Electronic Film Characterization

**Figure 1:**
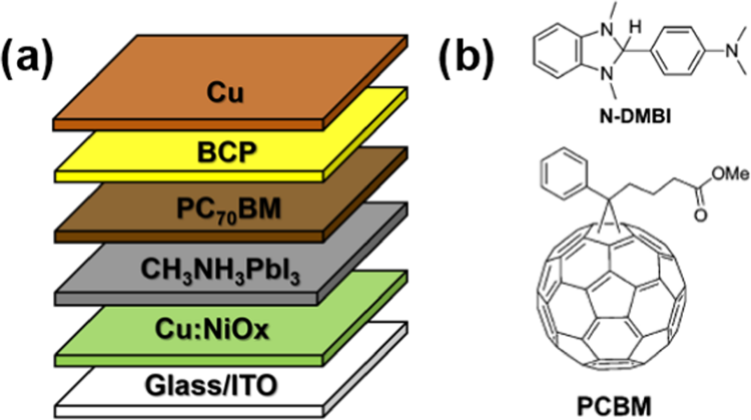
(a) Device architecture. (b) *N*-DMBI and fullerene schematic representation.

The fabricated devices were based on the p-i-n inverted device structure of ITO/Cu:NiO*_X_*/CH_3_NH_3_PbI_3_/PC_70_BM/BCP/Cu as shown in Figure 1a. The Cu:NiO*_X_* was coated using Dr. Blade coating, followed by combustion synthesis, as described in detail in Section 4.^26^ The perovskite active layer formulation was based on the solvent–antisolvent recipe used in our previous work and is described in detail in Section 4.^36,37^ The PC_70_BM was spin-coated on the active layer, followed by thermal evaporation of the BCP and Cu films.

**Figure 2:**
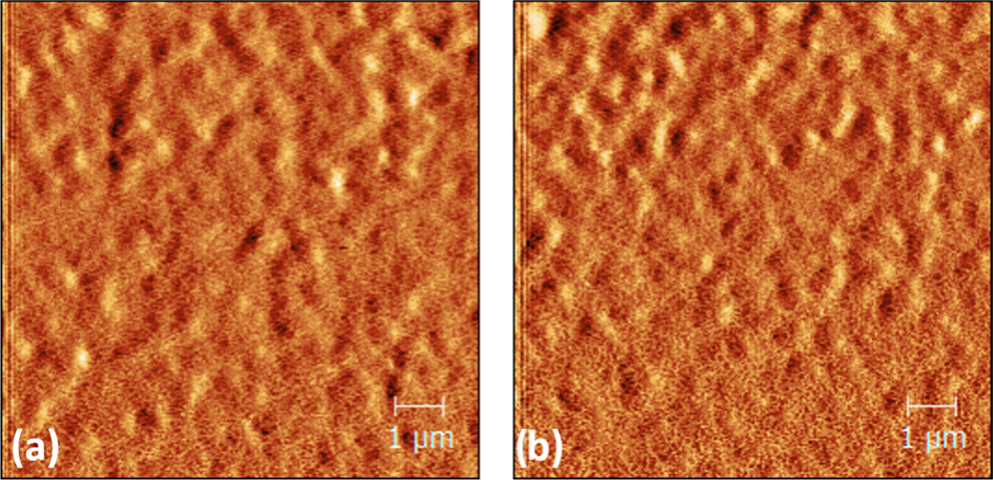
AFM measurements in phase contrast of (a) ITO/PC_70_BM pristine and (b) ITO/PC_70_BM (0.3% wt *N*-DMBI).

We fabricated ITO/PC_70_BM films to evaluate any potential morphological changes to the PC_70_BM due to doping. From the atomic force microscopy (AFM) data (Figure 2), we report similar surface topography between ITO/PC_70_BM undoped and 0.3% doped films (which is later shown to be the optimum doping concentration). The compactness of the films is similar between the two films while also retaining similar roughness values (1.34 nm for ITO/PC_70_BM undoped and 1.25 nm for 0.3% doped films).

### 2.2 Characterization of Pristine and *N*-DMBI-Doped PC_70_BM-Based Inverted Perovskite Solar Cells

**Figure 3:**
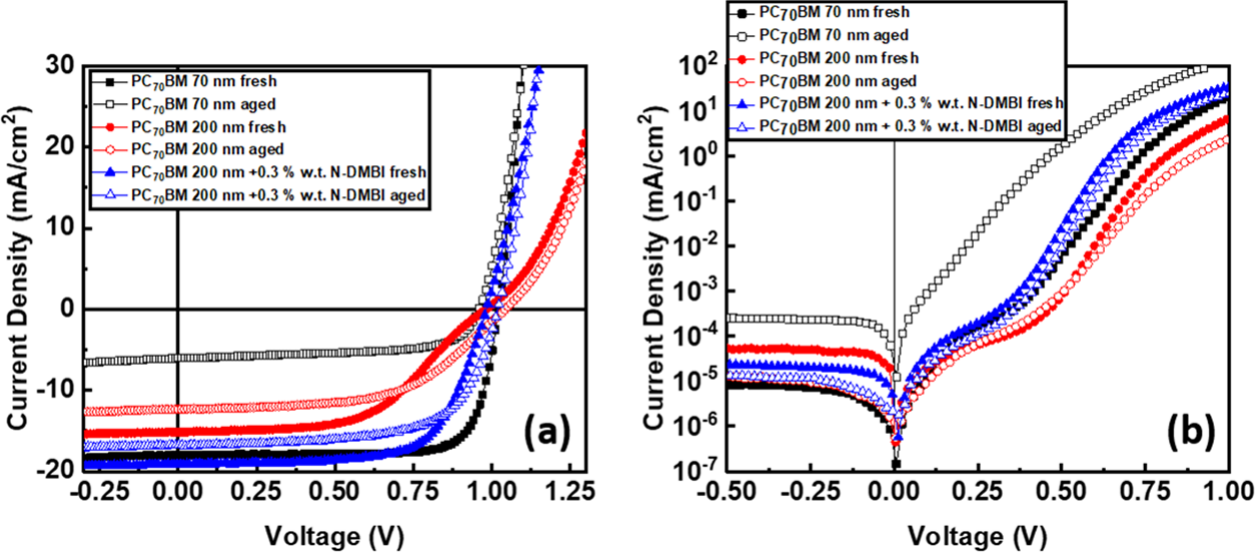
(a) Light J/V plots and (b) dark J/V plots of pristine and *N*-DMBI-doped PC_70_BM based devices.

To evaluate the effect of doping in devices with thick (200 nm) PC_70_BM ETLs, we have characterized both the light and dark J/V PV parameters when fresh as well as after aging at 60 °C on a hot plate inside a N_2_-filled glovebox. Various *N*-DMBI doping concentrations (0.1, 0.3, 0.5, and 0.7% wt) were evaluated to identify the optimum value for devices based on thick PC_70_BM ETLs. When we increase the thickness of PC_70_BM from 70 to 200 nm, we see a decrease of *V*_OC_ from 1.02 to 0.98 V, *J*_SC_ from 18.04 to 15.2 mA/cm^2^, FF from 78.5 to 52.5%, and PCE from 14.48 to 7.84% (Table 1), which is the same trend that we observed in our previous work.^18^ The experimental values of *J*_SC_ are in good agreement with the theoretically calculated *J*_SC_ values from the EQE data, which is <5% in margin of error (Figure S1). This is also shown in the light and dark J/V plots (Figure 3a,b). An increase in *R*_S_ from 4.9 to 13.1 Ω and a decrease in *R*_SH_ from 53.1 to 41.7 kΩ, which results in the decrease of *J*_SC_, *V*_OC_, and FF, respectively, due to the limited conductivity of PC_70_BM are observed. This is further discussed in the impedance spectroscopy characterization. By introducing the optimum n-type doping concentration of *N*-DMBI (0.3% wt), we managed to decrease *R*_S_ from 13.1 to 1.5 Ω and increase *R*_SH_ from 41.7 to 50.2 kΩ, which in return improve the *J*_SC_ from 15.2 to 19 mA/cm^2^, FF from 52.6 to 70.3%, and PCE from 7.84 to 13.1%. This improvement was reported in the literature to be due to the increase in conductivity and upshift of *E*_F_, leading to the enhancement of electron-transporting properties of PC_70_BM and an increase of the photocurrent of the device.^33,34^

**Table 1:** Perovskite Solar Cell Photovoltaic Parameters

device structure	*V*_oc_ (V)	*J*_sc_ (mA/cm^2^)	FF (%)	PCE (%)
ITO/Cu:NiO*_X_*/CH_3_NH_3_PbI_3_/PC_70_ BM (70 nm)/BCP/Cu	1.02	18.04	78.5	14.48
ITO/Cu:NiO*_X_*/CH_3_NH_3_PbI_3_/PC_70_BM (200 nm)/BCP/Cu	0.98	15.2	52.6	7.84
ITO/Cu:NiO*_X_*/CH_3_NH_3_PbI_3_/PC_70_BM (200 nm + 0.1% wt *N*-DMBI)/BCP/Cu	0.97	17.69	65.1	11.2
ITO/Cu:NiO*_X_*/CH_3_NH_3_PbI_3_/PC_70_BM (200 nm + 0.3% wt *N*-DMBI)/BCP/Cu	0.98	19	70.3	13.1
ITO/Cu:NiO*_X_*/CH_3_NH_3_PbI_3_/PC_70_BM (200 nm + 0.5% wt *N*-DMBI)/BCP/Cu	0.91	19.4	63	11.14
ITO/Cu:NiO*_X_*/CH_3_NH_3_PbI_3_/PC_70_BM (200 nm + 0.7% wt *N*-DMBI)/BCP/Cu	0.90	17.83	58.5	9.45

When the doping concentration is increased from 0.3 to 0.5 and especially 0.7 wt %, a drop in *V*_OC_ and FF is observed as shown in Table 1. Therefore, the optimum doping concentration was identified to be 0.3% wt. The statistical distribution of the PCE for the devices characterized in Figure 3a is shown in Figure S2. The J/V characterization for 0.1, 0.3, 0.5, and 0.7% wt. *N*-DMBI-doped devices is shown in Figure S3.

**Figure 4:**
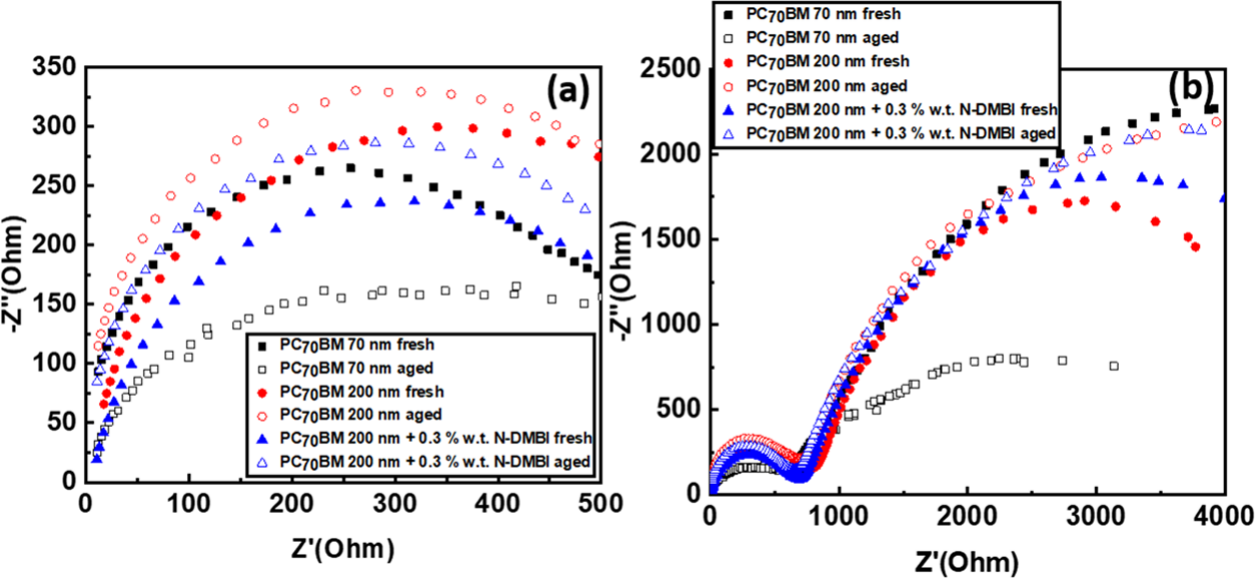
(a) Nyquist plot in high frequency and (b) Nyquist plot in low frequency.

Following the J/V characterization, we have performed impedance spectroscopy measurements to get more insight into the charge dynamics of the device. The impedance spectroscopy measurements presented in Figure 4 show two distinct frequency responses in the Nyquist plots, one at the high frequency (HF) range and one at the low frequency (LF) range. It has been previously reported for hybrid lead halide perovskite solar cells that the frequency response at HF is tied to the charge transport resistance (*R*_TR_) of the solar cells, whereas the LF response is tied to the charge recombination resistance (*R*_REC)_ of the solar cell.^38,39^

An increase in *R*_TR_ is observed when we increase the thickness of PC_70_BM (Figure 4a), which is in accordance with the increase of *R*_S_ from the dark J/V plots due to the limited conductivity of the material. Upon introducing 0.3% wt *N*-DMBI, we see a decrease in *R*_TR_ similar to the decrease in *R*_S_. Increasing the thickness of PC_70_ BM also reduces *R*_REC_ (Figure 4b), which is in accordance with the decrease of *R*_SH_. *R*_REC_ is tied to the charge accumulation under illumination^37^ at the electrodes of the device. Using thick PC_70_BM, we introduce more charge recombination events in the ETL due to the increased trapping of the thick film and its limited conductivity. *R*_REC_ is substantially improved upon introducing 0.3% wt. *N*-DMBI, which can also be seen from the improvement of the FF, however, is still lower than the thin PC_70_BM-based devices.

### 2.3 Accelerated Lifetime Performance and Simulation Studies of Pristine and *N*-DMBI-Doped PC_70_BM Diffusion Blocking Layer-Based Inverted Perovskite Solar Cells

**Figure 5:**
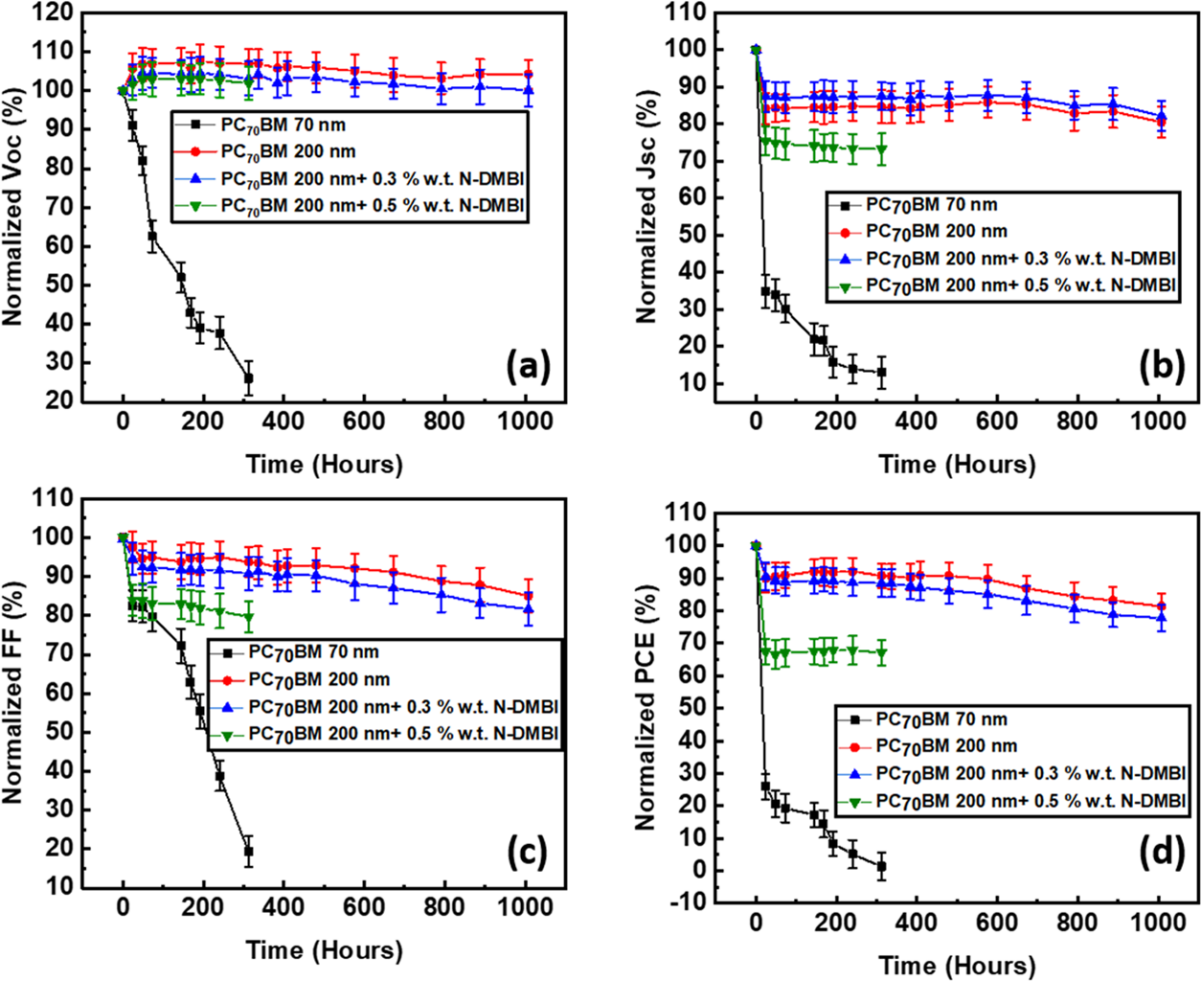
Normalized lifetime plots of (a) *V*_OC_, (b) *J*_SC_, (c) FF, and (d) PCE after aging in 60 °C, N_2_.

Following the characterization of 0.3% wt *N*-DMBI-based devices, we have performed accelerated heat lifetime tests (60 °C, N_2_) to verify that the devices based on thick-doped PC_70_BM ETL not only had improved PV parameters but also retained a high lifetime. The goal of this work was not to achieve the highest possible PCE but to evaluate the application of thick fullerene diffusion blocking layers in conjunction with just enough doping to achieve high stability while still retaining a good compromise between the lifetime and PV performance. The *J*_SC_ trend is also visualized using photocurrent mapping (PCT) between fresh and aged devices. From the lifetime plots of Figure 5, we see that in our reference devices based on thin (70 nm) PC_70_BM ETL, there is a rapid drop in all PV parameters even at the first 24 h of aging. This drop in PCE has been previously identified to be a result of ion diffusion (especially I^–^) toward the electrodes of the device.^18^ By increasing the thickness of PC_70_BM, the lifetime is improved, by functioning as a thick diffusion blocking layer, achieving *T*_80_ at 1000 h, however, as discussed previously in this report, this comes at an expense to the PCE of the device due to the limited conductivity of PC_70_BM. Using 0.3% wt *N*-DMBI doped devices, we report improved lifetime while still retaining a good PCE. It is important to note that when the doping was increased from 0.3 to 0.5% wt *N*-DMBI, there was also a significant drop in efficiency at early times.

**Figure 6:**
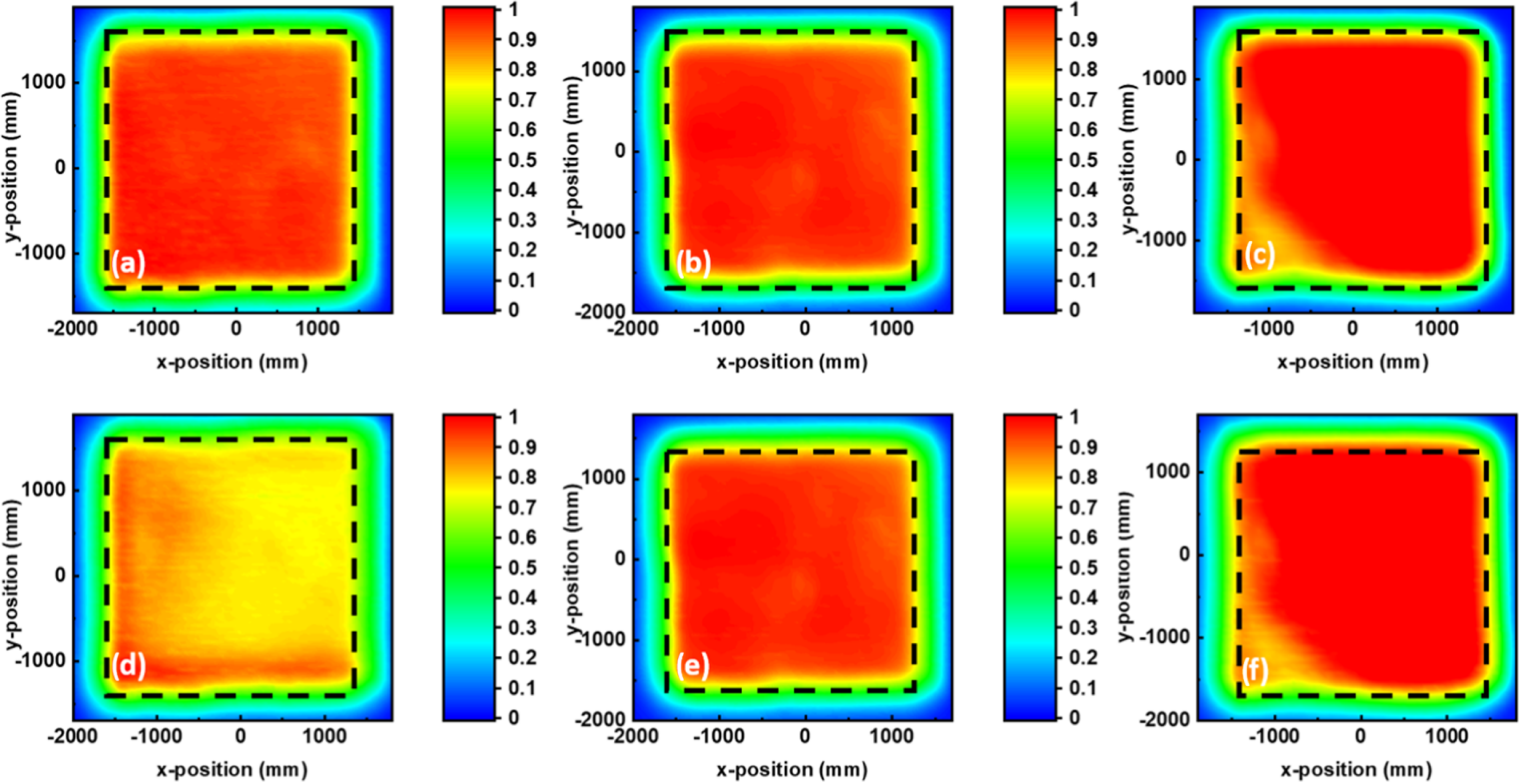
PCT data of PC_70_BM-based devices: (a) 70 nm fresh, (b) 200 nm fresh, (c) 200 nm 0.3% wt *N*-DMBI fresh, (d) 70 nm aged, (e) 200 nm aged, and (f) 200 nm 0.3% *N*-DMBI aged.

To visualize the trend of *J*_SC_ lifetime, we have performed (PCT) measurements for all of the devices evaluated. The results are presented in Figure 6. As shown, the trend of *J*_SC_ closely follows the PCT data. It is important to note that there is a slight increase in *V*_OC_ upon aging for the devices based on thick (200 nm) doped and undoped PC_70_BM-based devices. It has been previously reported that heat treatment can promote PC_70_BM diffusion toward the grain boundaries of the perovskite active layer, assisting in their passivation.^40^ Intentional doping of the perovskite active layer with PC_70_BM has been reported to improve the PV parameters of PSCs,^41^ and we believe that in our case, a similar mechanism can happen due to a combination of large PC_70_BM thickness and its promoted diffusion due to the accelerated heat tests. This is further highlighted by the increase in *R*_SH_ from the dark J/V plots and *R*_REC_ from the Nyquist plots, which point toward reduced charge recombination, which can happen upon grain boundary passivation.

**Figure 7:**
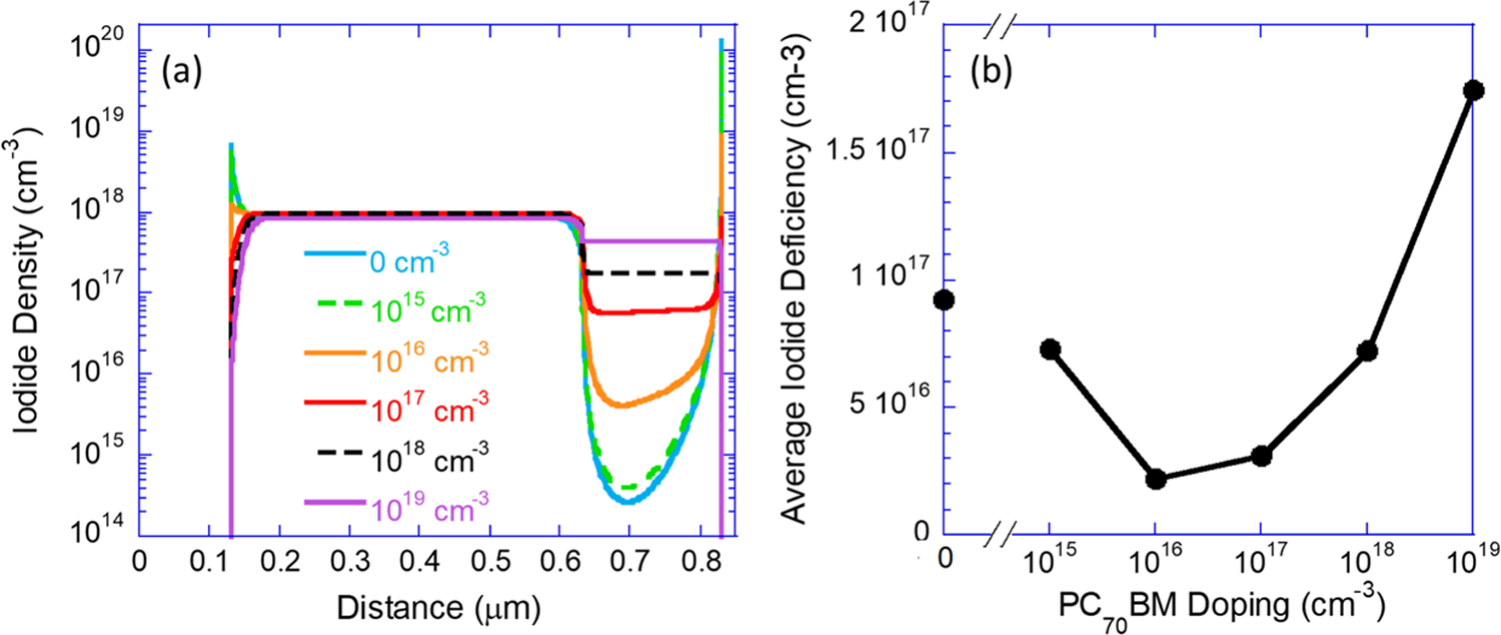
(a) Simulated iodide density distribution within the PSC for different doping levels of the electron transport layer (ETL). (b) Average iodide deficiency in the perovskite layer. The simulations were performed under 1 Sun and a bias close to the maximum power point.

To better understand the origin of an optimal doping level, we performed simulations similar to those reported in ref (35), with the device structure reported here. In short, we use a semiconductor device simulator by synopsis solving the drift-diffusion and Poisson equations for electrons, holes, and anions (iodide). The cations are taken to be static within the perovskite layer, and the initial ions’ density within the perovskite layer was 10^18^ cm^–3^. Figure 7a shows the iodide density distribution at a steady state under 1 Sun and a bias close to the maximum power point. The color coding marks the density of ionized N-type dopants at the electron transport layer (ETL). Due to noncomplete ionization, the actual dopant density could be significantly higher. The figure shows that the iodide penetrates the ETL and may accumulate at the contact interface. At low to no doping, the iodide is primarily close to the contact interface. As the doping density increases, the ETL is filled with iodide, while the density at the contact interface reduces. The trend shown in Figure 7a indicates that there should be an optimum doping density, and we show in Figure 7b the average deficiency of the iodide density in the perovskite layer as a function of the density of ionized dopants. This subfigure shows that there is an optimum at 10^16^ to 10^17^ cm^–3^ ionized dopants in the PC_70_BM layer.

The combined experimental and simulation results presented above show a clear correlation between PC_70_BM n-type doping and iodide distribution and experimentally verified the importance of just enough doping principle on the PSC lifetime performance.

## 3 Conclusions

In conclusion, we have shown that thick PC_70_BM ETLs can be successfully utilized as diffusion blocking layers for p-i-n inverted PSCs. By utilizing an optimal n-type doping concentration of 0.3% wt *N*-DMBI in conjunction with thick (200 nm) PC_70_BM ETLs, we have achieved highly stable (*T*_80_ = 1000 h under 60 °C, N_2_) devices while still retaining relatively good PCE at 13.1%. By increasing the doping from 0.3 to 0.5 wt %, we observed a drop in the initial efficiency as well as enhanced degradation. With the aid of the simulations, we correlate the experimental results with the penetration of iodide into the blocking layer and the corresponding iodide deficiency created in the perovskite photovoltaic active layer. Retaining a good PCE was mainly a result of improved *J*_SC_ and FF due to the improvement of the PC_70_BM conductivity, which resulted in a decrease of *R*_S_ from 13.1 to 1.5 Ω and an increase of *R*_SH_ from 41.7 to 50.2 kΩ. Utilizing a thick PC_70_BM diffusion blocking layer while still retaining a good compromise between the lifetime and PCE is important for the up-scalability of p-i-n- inverted PSCs and just enough doping is essential to retain this balance. The optimum doping density was correlated with the deficiency of iodide density in the perovskite layer through simulations. The proposed method of incorporating a 0.3% wt *N*-DMBI-doped thick fullerene diffusion blocking layer within the inverted PSC device architecture can be in practice easily applied to the roll-to roll printing manufacturing process providing a simple device engineering route for achieving high-performance PSCs.

## 4 Experimental Methods

### 4.1 Materials

Toluene (99.7%), nickel(II) nitrate hexahydrate (>98.5%) (Ni(NO_3_)_2_·6H_2_O), copper(II) nitrate trihydrate (99–104%) (Cu(NO_3_)_2_·3H_2_O), 2-methoxyethanol anhydrous (99.8%), acetylacetone (≥99%), dimethyl sulfoxide (≥99.7%),γ-butyrolactone (≥99%), chlorobenzene anhydrous (99.8%), and 4-(2,3-dihydro-1, 3-dimethyl-1*H*-benzimidazol-2-yl)-*N*,*N*-dimethylbenzenamine (98%) were purchased from Sigma-Aldrich Chemicals. Methylammonium iodide (≥99%) was purchased from GreatCell Solar. Lead(II) iodide (99.999%) and bathocuproin (98%) were purchased from Alfa Aeser. Phenyl-C70-butyric acid methyl ester (99%) was purchased from Solenne BV. Cu pellets were purchased from Kurt J. Lesker. Ultrapure water was produced by a milli-Q Academic system, Millipore (Burlington, MA). All solutions were prepared with analytical grade chemicals and ultrapure milli-Q water with a conductivity of 18.2 μS/cm. ITO-patterned glass substrates (sheet resistance 4 Ω/sq) were purchased from Psiotec Ltd.

### 4.2 Cu:NiO*_x_* Solution

For the combustion synthesis of Cu:NiO_*x*_, 0.95 mmol of Ni(NO_3_)_2_·6H_2_O and 0.05 mmol of Cu(NO_3_)_2_·3H_2_O were dissolved in 2.5 mL of 2-methoxyethanol. The solutions were stirred at 50 °C for 1 h under ambient conditions, and then, 0.1 mmol of acetylacetone was added to the solution, and the whole solution was left for further stirring for 1 h at room temperature.

### 4.3 Perovskite Solution

The perovskite solution was prepared using a mixture of MAI/PbI_2_ (1:1). The mixture was dissolved in a solvent of γ-butyrolactone/DMSO (7:3). The solution was stirred at 60 °C for 1 h in an inert atmosphere inside a N_2_-filled glovebox. The perovskite solution was left to cool at room temperature inside the glovebox followed by filtering using a 0.22 μm PVDF filter.

### 4.4 PC_70_BM Solution

The PC_70_BM solution was prepared at concentrations of 20 mg/mL (for 70 nm films) and 50 mg/mL (for 200 nm films) in chlorobenzene. The solution was left overnight at 60 °C under ambient conditions.

### 4.5 *N*-DMBI Solution

*N*-DMBI solutions of various concentrations to achieve the required doping concentrations were prepared in CB and left overnight under stirring and at 60 °C under ambient conditions. The solutions were then mixed with the PC_70_BM solution the next day.

### 4.6 Device Fabrication

ITO-patterned glass substrates were cleaned using an ultrasonic bath for 10 min in acetone followed by 10 min in isopropanol. The Cu:NiO*_x_* films (30 nm) were coated using a Doctor Blade with a blade speed of 5 mm/s and a plate temperature of 85 °C. The films were annealed at 300 °C on a hot plate for 1 h in ambient atmosphere. The perovskite films were coated inside a N_2_ atmosphere glovebox using a three-step spin-coating process: first step, 500 rpm for 5 s; second step, 1000 rpm for 45 s; and third step, 5000 rpm for 45 s. During the third step, after the first 20 s of the duration of the step, 0.5 mL of toluene was dropped onto the spinning substrate as the antisolvent to achieve the rapid crystallization of the films. The resulting perovskite films (300 nm) were annealed at 100 °C for 10 min. The PC_70_BM film (70 and 200 nm depending on the concentration) was coated inside the glovebox by using spin coating at 1300 rpm for 30 s. The BCP (7 nm) and Cu layers (80 nm) were deposited using thermal evaporation.

### 4.7 Device Characterization

The thicknesses and surface profile of the device layers were measured with a Veeco Dektak 150 profilometer, Hong Kong, China. The current density–voltage (J/V) characteristics were characterized with a Botest LIV Functionality Test System (Kreuzwertheim, Germany). Both forward (short circuit–open circuit) and reverse (open circuit–short circuit) scans were measured with 10 mV voltage steps and 40 ms of delay time. For illumination, a calibrated Newport Solar simulator (Irvine, CA) equipped with a Xe lamp was used, providing an AM1.5G spectrum at 100 mW/cm^2^, as measured by a certified Oriel 91,150 V calibration cell. A custom-made shadow mask was attached to each device prior to the measurements to accurately define the corresponding device area (9 mm^2^). EQE measurements were performed by Newport System (Irvine, CA), Model 70356_70316NS. Impedance spectroscopy was performed using a Metrohm Autolab PGSTAT 302N equipped with the FRA32 M module (Herisau, Switzerland). To extract the Nyquist plots, the devices were illuminated using a white LED. A small AC perturbation voltage of 10 mV was applied, and the current output was measured at a frequency range of 1 MHz to 1 Hz. The steady-state DC bias was kept at 0 V. Atomic force microscopy (AFM) data was obtained using a Nanosurf easyScan 2 controller in tapping mode. Photocurrent (PCT) measurements were performed using a Botest Photoelectric Test System—PCT 1 equipped with a 405 nm laser at 25 mW power. The devices were taken outside the glovebox to measure their PV parameters and undergo device characterization at 24 h intervals as needed. The devices were placed back in the glovebox to continue the aging process every 24 h.

## Supporting Information Available

EQE data for thin (70 nm), thick (200 nm), and doped (0.3% wt *N*-DMBI) fresh and aged devices (Figure S1); statistical distribution of PCE for devices based on (a) PC_70_BM 70 nm, (b) PC_70_BM 200 nm, and (c) PC_70_BM 200 nm + 0.3% wt *N*-DMBI (Figure S2); and illuminated J/V characteristics of devices based on 0.1, 0.3, 0.5, and 0.7% wt *N*-DMBI doping (Figure S3) (PDF)
