# Supplementary material for: Optimized Doping of Diffusion Blocking Layers and Their Impact on the Performance of Perovskite Photovoltaics
Source: ACS Appl Electron Mater. 2023 Oct 12;5(10):5580–7. doi: 10.1021/acsaelm.3c00900 (PMC10601534; doi:10.1021/acsaelm.3c00900)
Supplement: Supplementary file 1 — el3c00900_si_001.pdf [file el3c00900_si_001.pdf]

Supporting Information

Optimized Doping of Diffusion Blocking Layer and its Impact on the Performance of Perovskite Photovoltaics

Fedros Galatopoulos [1], Sapir Bitton [2], Maria Tziampou [1],

*Nir Tessler [2] and Stelios A. Choulis [1]**

[1] Molecular Electronics and Photonics Research Unit, Department of Mechanical Engineering and Materials Science and Engineering, Cyprus University of Technology, Limassol, 3603, Cyprus.

[2] Sara and Moshe Zisapel Nano-Electronic Center, Department of Electrical Engineering, Technion-Israel

Institute of Technology, Haifa 32000, Israel

* Corresponding Authors: stelios.choulis@cut.ac.cy

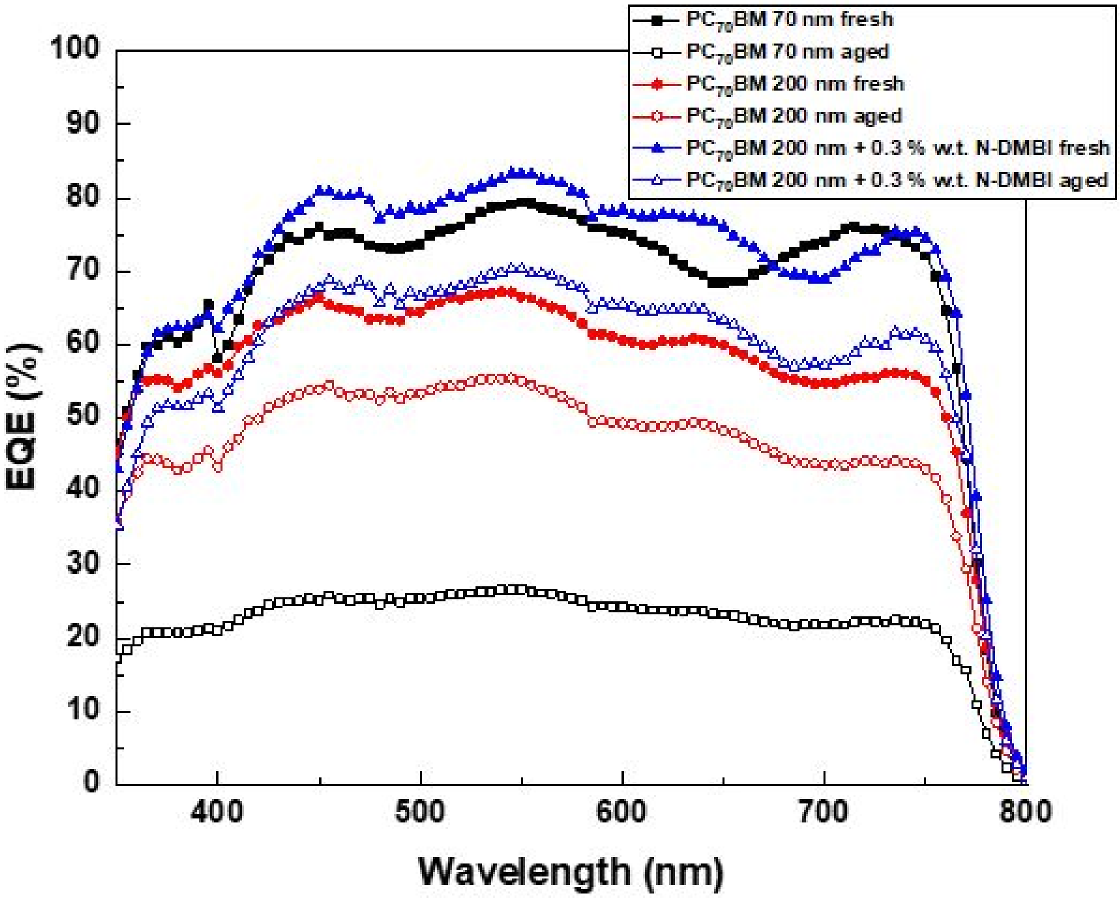

Figure S1: EQE data for thin (70 nm), thick (200 nm) and doped (0.3 % w.t. N-DMBI) fresh and aged devices

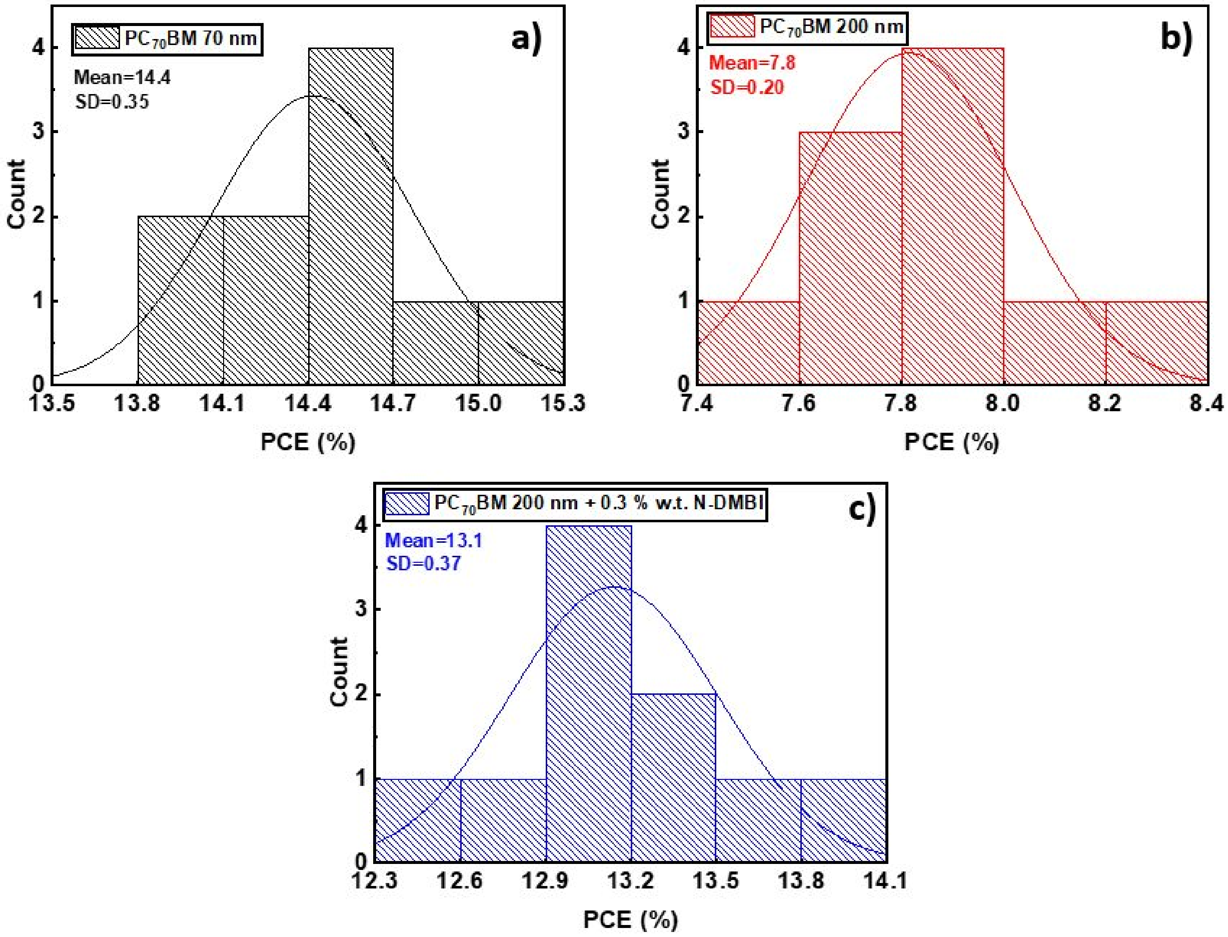

Figure S2: Statistical distribution of PCE for devices based on: a) $PC_{70}BM$ 70 nm, b) $PC_{70}BM$ 200 nm and c) $PC_{70}BM$ 200 nm + 0.3 % w.t. N-DMBI

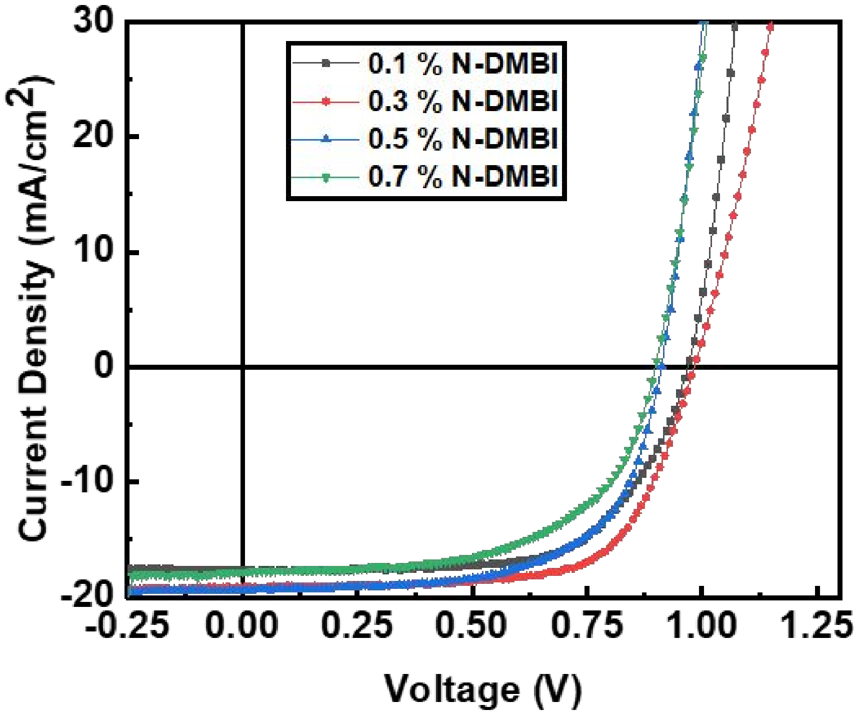

Figure S3: Illuminated J/V characteristics of devices based on 0.1, 0.3, 0.5 and 0.7 % w.t. N-DMBI doping
